# COVID-19, India, lockdown and psychosocial challenges: What next?

**DOI:** 10.1177/0020764020935922

**Published:** 2020-06-13

**Authors:** Mahaveer Golechha

**Affiliations:** Indian Institute of Public Health Gandhinagar, Gandhinagar, India

A world pandemic threat COVID-19 mitigation is crucial to the human life and for reducing distortion of livelihood. The COVID-19 pandemic has swept into more than 200 countries with considerable confirmed cases and deaths and has caused public panic and mental health stress (Huang & Zhao, 2020). Most of the nations across the world have implemented complete lockdown with stringent social distancing measures for breaking the chain of transmission. The current outbreak of COVID-19 is heavily impacting the global health and mental health. Despite all resources employed to counteract the spreading of the virus, additional global strategies are needed to handle the related mental health issues (Torales et al., 2020). This outbreak is leading to additional health problems such as stress, anxiety, depressive symptoms, insomnia, denial, anger and fear globally (Torales et al., 2020). To protect people and prevent the spread, it is critical that public mental health paradigms and measures are used (Ventriglio et al., 2020).

On 30 January 2020, India reported first case of COVID-19 and the numbers have risen steadily since then, albeit at an alarming rate in the final days of March. Aiming to control community transmission, the world’s largest democracy has implemented world’s largest nationwide lockdown since 24 March 2020 (The Lancet, 2020). The country remains vulnerable towards COVID-19, given the high population density, socioeconomic fabric and overstretched health-care infrastructure.

The total lockdown was the only immediately available, best and ideal solution to the control COVID-19 pandemic in India. The Indian government has responded appropriately, adequately and quickly to the COVID-19 pandemic at multiple levels. The lockdown has helped India in buying crucial time: time for extensive contact tracing, time to ramp up testing and most crucially, time to prepare our health system, increasing its health-care infrastructure and preventing it from overwhelming, as it happened in Italy, the United States and Spain.

The lockdown is an effective strategy for containing the spread of infection. However, this is very challenging with added difficulty for larger sections of the society. The social distancing is very difficult for many households in India, especially slum areas; the daily-wage earner has to earn daily money to keep family alive, and people with existing mental health illnesses face severe issues. A long-time lockdown may lead to psychosocial difficulties for vulnerable population and consequently lead to stress, anxiety, frustration, boredom and depression and even suicidal idea and attempts. The Lancet Psychiatry (2020) also highlighted the mental health needs of vulnerable groups, including those with severe mental illness, learning difficulties and neurodevelopmental disorders, as well as socially excluded groups such as prisoners, the homeless and refugees. Nevertheless, the burden of this infection on the global mental health is currently neglected even if it may challenge patients, general population as well as policy makers and health organisations and teams (Torales et al., 2020).

India’s health inequalities, flaring economic and social disparities and distinct cultural values had made lockdown a hard measure for the poorer sections of the society. The nationwide lockdown has maximised economic loss and simply debilitated the country’s large population of daily-wage earners and migrant labourers and become an important mental health problem. The emerging mental health issues related to this global event may evolve into long-lasting health problems, resulting in isolation and stigma for vulnerable population in the country.

The extended lockdown will lead to economic hardship, famine, psychosocial challenges and law and order issues, which may in turn undermine benefit gauge by lockdown and COVID-19 containment objectives. In Indian settings, this may exacerbate health inequalities and reinforce the vicious cycle between poverty and ill health.

The social and economic issues due to COVID-19 pandemic will result in mass unemployment, depleted social safety nets, homelessness, increase in gender-based violence, alcoholism, hunger, loan defaults and millions slipping into poverty. This post-COVID landscape will definitely leads to an increase in mental health issues such as chronic stress, anxiety, depression, alcohol dependence and self-harm. Recent evidences in psychosocial sciences also show that similar pandemics increased the prevalence of symptoms of post-traumatic stress disorder (PTSD), as well as confusion, feeling of loneliness, boredom and anger during and after quarantine (Brooks et al., 2020).

The Ministry of Health and Family Welfare, Government of India has taken several steps to deal with mental health challenges posed by COVID-19, which includes development of various guidelines in collaboration with National Institute of Mental Health and Neuroscience. The guidelines aimed at enhancing resilience of vulnerable populations against mental health issues. The Ministry of Health and Family Welfare has also established helpline for behavioural and psychosocial help.

However, a lot needs to be done, including capacity building of frontline health-care worker and a large-scale public engagement campaign to increase help-seeking, creating and spreading awareness through mainstream media and social media giants. The real need is to build community-based capacity to handle local issues long after the acute phase of the epidemic. A small team of peer counsellors work under a local administrator and trained on community mental health issues.

It is time to build mental wellbeing and resilience into schools, the community and their families. We need a systemic approach to build the demand for mental wellbeing. The Government of India’s Rashtriya Kishor Swasthya Karyakram (National Adolescent Health Programme) can play a pivotal role in social and behavioural change and enhance adolescent resilience against mental health challenges posed by the pandemic.

Furthermore, the government should give special attention to systematic psychological health care which is required by health-care staff and patients, and systematic psychological self-care must be given a high priority in coping with the detrimental impacts of COVID-19 and social distancing (Matias et al., 2020).

The nationwide lockdown has proven as a successful strategy for India, and it has also helped in containing the spread of COVID-19 across various states. The lockdown has already achieved the desired effect of flattening the epidemic curve (The Lancet, 2020). Therefore, it is right time for India to plan quick gradual, phased and calibrated withdrawal strategy. A well-planned calibrated multi-phase exit strategy will be required post lockdown.

India can end the lockdown now and additional revenue available from the revival of the economy can be spend on increasing testing, isolation facilities, hospital beds, critical care and comprehensive Information, Education and Communication (IEC) on social distancing and mental health and addressing the mental health issues of vulnerable population post lockdown. The country can gain much more through continuation of bans on mass gatherings, school closure, restriction of movement of elderly population and children below the age of 5 years, covering mouths and noses in public, spitting bans, physical distancing to the extent possible in public places and expanded testing. Mental health support and follow-up should be provided even 6 months after the release from isolation for those individuals with prior vulnerable mental health status.

Meanwhile, India should increase the testing. When we look at the rate which screening is being done, India ranks at the lower end of the spectrum. The country needs ‘exponential’ ramping up of testing to leverage the benefit provided by nationwide lockdown. The panacea against prolonged lockdown and for reassuming economic activity is large-scale testing.

The importance of community involvement, awareness and behaviour change cannot be undermined in the current situation, especially for psychosocial issues due to COVID-19. Risk communication and community engagement is a critical component of the response to COVID-19 (World Health Organization (WHO), 2020). This crisis is not going to be controlled without community participation because ultimately control is based on individual behaviour. The Government should take various measures like behavioural change communication, hand washing facilities and improving availability and accessibility of community-based mental health services. Community psychological interventions and support might have some effects in reducing PTSD symptoms and depressive and anxiety symptoms in adults during these stressful events.

This is an opportunity for India to recognise the importance of strong public health systems and increasing investment in health for making its health system resilient towards future pandemic. The governments need to step up to protect their populations and people in a non-threatening, non-panicky manner to ensure safety of all individuals. The country should focus more on improving primary care, health-care infrastructure and human resources for health. India’s public health-care system is chronically underfunded (at just 1.5% of GDP; Chetterje, 2020), leaving primary care weak. This pandemic could be the much-needed wake-up call to the necessity of long-term changes to India’s health system.
